# Microenvironment, Oncoantigens, and Antitumor Vaccination: Lessons Learned from BALB-neuT Mice

**DOI:** 10.1155/2014/534969

**Published:** 2014-06-03

**Authors:** Laura Conti, Roberto Ruiu, Giuseppina Barutello, Marco Macagno, Silvio Bandini, Federica Cavallo, Stefania Lanzardo

**Affiliations:** Department of Molecular Biotechnology and Health Sciences, Molecular Biotechnology Center, University of Torino, Via Nizza 52, 10126 Torino, Italy

## Abstract

The tyrosine kinase human epidermal growth factor receptor 2 (*HER2*) gene is amplified in approximately 20% of human breast cancers and is associated with an aggressive clinical course and the early development of metastasis. Its crucial role in tumor growth and progression makes HER2 a prototypic oncoantigen, the targeting of which may be critical for the development of effective anticancer therapies. The setup of anti-HER2 targeting strategies has revolutionized the clinical outcome of HER2^+^ breast cancer. However, their initial success has been overshadowed by the onset of pharmacological resistance that renders them ineffective. Since the tumor microenvironment (TME) plays a crucial role in drug resistance, the design of more effective anticancer therapies should depend on the targeting of both cancer cells and their TME as a whole. In this review, starting from the successful know-how obtained with a HER2^+^ mouse model of mammary carcinogenesis, the BALB-neuT mice, we discuss the role of TME in mammary tumor development. Indeed, a deeper knowledge of antigens critical for cancer outbreak and progression and of the mechanisms that regulate the interplay between cancer and stromal cell populations could advise promising ways for the development of the best anticancer strategy.

## 1. Introduction

**Figure 1:**
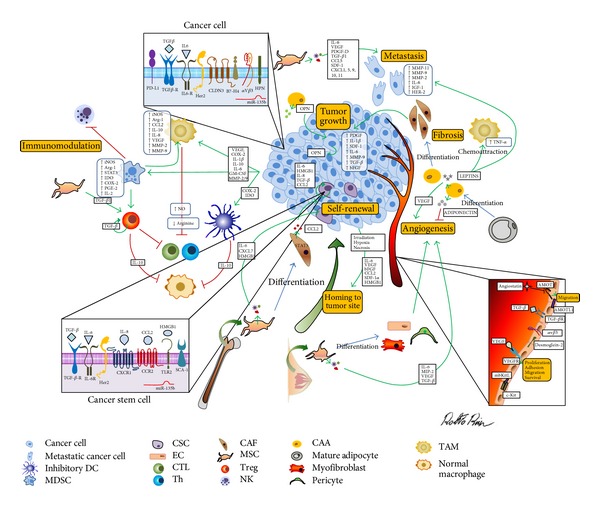
Interconnections between the population present in TME in breast tumors. Elevated levels of cytokines and growth factors produced by tumor and stromal cells orchestrate tumor development and progression. Abbreviations: mesenchymal stem cell (MSC), endothelial cell (EC), cancer-associated fibroblast (CAF), cancer stem cell (CSC) adipocyte (CAA), dendritic cell (DC), natural killer (NK), regulatory T (Treg) cell, myeloid derived suppressor cell (MDSC), tumor associated macrophages (TAMs), cytotoxic T lymphocytes (CTL), T helper (Th), interleukin (IL), toll-like receptor (TLR) 2, high mobility group box (HMGB) 1, vascular endothelial growth factor (VEGF), matrix metalloproteinase (MMP), stromal cell-derived factor- (SDF-) 1, transforming growth factor- (TGF-)*β*, chemokine (C-C motif) ligand (CCL)2, angiomotin (Amot), angiomotin-like (AmotL) 1, membrane-bound KitL (mbKitL), tumor necrosis factor- (TNF-)*α*, interferon- (IFN-)*γ*, nitric oxide synthase (iNOS), arginase (Arg) 1, indoleamine-2, 3-dioxygenase (IDO), cyclooxygenase- (COX-) 2, transcription factor signal transducer and activator of transcription (STAT) 3, programmed death (PD) 1, osteopontin (OPN), prostaglandin E- (PGE-) 2, platelet-derived growth factor (PDGF), macrophage inflammatory protein- (MIP-) 2, fibroblast growth factor (FGF), Insulin-like growth factor- (IGF-) 1, and tyrosine kinase human epidermal growth factor receptor (HER) 2.

Solid tumors are currently considered to be organ-like structures, composed of cancer cells and other cells that support tumor development. While deep understanding of cancer cells has been reached, less light has been shed on the cell populations that make up the tumor microenvironment (TME), as they have been ostracized for several decades and are only now being reappraised as a driving force for tumor pathogenesis. TME is composed of cells—such as inflammatory cells, mesenchymal stem cells (MSCs), endothelial cells (ECs), cancer-associated fibroblasts (CAFs), and adipocytes (CAAs)—and soluble factors, cytokines, and the extracellular matrix (Figure 1) that bidirectionally communicate with cancer cells. This continuous and finely tuned interplay can promote cancer outbreak, sustain tumor development and invasion, defend a tumor from host immunity, foster therapeutic resistance, and provide niches for cancer stem cells (CSCs) and dormant metastases [1]. In this respect, TME is now considered to be a good target for anticancer therapies, as it provides the opportunity to perturb the delicate balance that promotes tumor progression. In fact, similarly to tumor cells [2], TME is now thought of as the source of a broad range of targets, of which the most promising are tumor-associated antigens that play a key role in cancer development and progression, called oncoantigens (OAs) [3]. We have recently classified OAs according to cellular localization [4]: Class I (cancer cell surface antigens), Class II (soluble antigens and antigens expressed in the TME), and Class III (intracellular proteins expressed by cancer cells). They are currently emerging as ideal targets for a very specific anticancer treatment, as demonstrated by several studies in preclinical models [3].

HER2 represents the prototypic Class I OA and is found to be overexpressed in a variety of human cancers [5]. HER2 amplification or overexpression is found in 15–20% of all new breast cancer cases and is a prognostic marker of poor outcome [6]. Currently, the identification of HER2 positivity in tumor tissue specimens allows for patient stratification and a more reasonable therapeutic strategy. Indeed, a number of tyrosine kinase inhibitors or monoclonal antibodies (mAbs) that are directed against HER2 are available, while others are currently under investigation in several phase I to III clinical trials [7]. Humanized mAb Trastuzumab is the standard of care in breast cancer treatment in both preoperatory and metastatic settings, whether it is used as a single agent or in association with chemotherapy. Despite initial responsiveness, the majority of patients that suffer from either primary or metastatic breast cancer develop drug resistance within one year, rendering Trastuzumab completely ineffective [8]. Similarly, prolonged exposition to anti-HER2 tyrosine kinase inhibitors often results in the development of HER-2-negative tumor variants [9]. The mechanisms that underlie primary and acquired resistance to HER2-targeted therapies are still under investigation. However, both CSCs and TME seem to play a crucial role in these phenomena [10]. This fact emphasizes the need to consider cancer cells and their TME as a whole when designing effective anticancer therapies and tells us that targeting a single OA is not sufficient to freeze tumor progression, a possibility that can only be explored thanks to the availability of appropriate* in vivo* cancer models.

The identification of appropriate murine models that are able to mimic most of the features of a human cancer offers considerable potential to give advantages in the race towards the clinic. In particular, the availability of tumor-transplantable models and genetically engineered mammary cancer-prone mice has allowed laboratories to decipher the most important mechanisms involved in mammary tumor development and progression, thus permitting current therapies to be refined. A great deal of data has been obtained by our group from transgenic mice, called BALB-neuT, that overexpress the rat HER2 (neu) oncogene under the mouse mammary tumor virus (MMTV) promoter [11], with this very fact in mind. These mice spontaneously develop mammary carcinomas with 100% penetrance [12] and display a histopathologically [13] and transcriptionally [14] well characterized course that closely recapitulates many features of human breast carcinogenesis. In virtue of the high homology of BALB-neuT tumors to human HER2 positive breast cancer, this is an ideal model to use when setting up new anticancer therapies. Actually, BALB-neuT mice and the cell line derived from a BALB-neuT adenocarcinoma (TUBO cells) have provided us with a fascinating tool and one that is used in many laboratories worldwide to deepen current knowledge of the pathogenic mechanisms that promote HER2 positive tumor growth and consequently elaborate more efficacious antitumor strategies. We herein discuss the lessons learned about TME, HER2, and other OAs from BALB-neuT mice and how this knowledge can help develop a winning strategy against cancer.

## 2. The Urgency of Defining the Most Promising TME-Associated OAs

Neoplastic transformation is a multistep process which involves specific proteins and regulatory pathways at each stage. The identification of the genes that constitute the driving force of cancer progression is an extraordinary opportunity to gain an advantage over cancer. HER2 represents a paradigm of this conception; its expression at the neoplastic stage, its overexpression in established tumors, and its causal role in cancer progression [14] make it the ideal immunological target. This observation has paved the way for the development of new immunologically based therapies against neoplastic cells that overexpress HER2, which have made some important clinical achievements [15]; the U.S. Food and Drug Administration (FDA) has approved mAbs that target HER2, such as Trastuzumab and Pertuzumab, and several drugs (i.e., TDM1 and ARRY-380) [16], which have prolonged the disease-free survival rates in patients with metastatic HER2 positive breast cancer [17] and are currently under investigation in clinical trials. However, the majority of patients treated with these agents develop resistance within one year of treatment, resulting in disease progression, recurrence, and reduced overall survival [18]. Similar results have also been obtained using active immunotherapy against HER2 in preclinical models [19]. The efficacy of DNA vaccines targeting HER2 in BALB-neuT mice [20] relies mostly on the direct activity of vaccine-elicited Abs [21–23] and is strictly dependent on the tumor stage at the time of vaccination; the sooner the vaccination is performed, the better the outcome [24]. When the vaccine is administered to a still healthy BALB-neuT mouse, repeated boosts keep it tumor free for a period of time that may well equate to its natural life span. However, when the same vaccine is administered to a mouse in a more advanced stage of microscopic lesions, the appearance of palpable tumors is only slightly delayed. This suggests that targeting a single oncoantigen is not sufficient to freeze tumor progression, especially when it is applied to patients that suffer from advanced cancer, as commonly happens in the clinical setting [12].

This partial failure of anti-HER2 treatment suggests that some key elements that drive mammary carcinogenesis must still be sought out and not only on the tumor cells themselves; the best chance of defeating cancer that we have is offered by targeting both cancer cells and TME. TME can dynamically control cancer progression thanks to its continuous interplay with cancer cells [25]. Therefore, the identification of additional OAs that are expressed by either tumor or stromal cells surrounding HER2 positive lesions is urgently needed if we are to develop a combined and more efficient anticancer approach which may prevent the development of the very resistance to anti-HER2 therapy that is responsible for tumor relapse [26].

To address this point, we performed a transcription profile analysis of BALB-neuT preneoplastic and invasive lesions, integrated with a meta-analysis of data obtained from healthy human and neoplastic specimens. Of the 46 putative OAs identified [27], B7-H4 [28], Claudin 3 [29], Hepsin [30], CD52 [31], and Desmoglein 2 [32] are Class I OAs, expressed on the plasma membrane of cancer and TME cells and therefore constitute promising targets for vaccination. Class II OAs are another group of identified OAs and includes cytokines and chemokines copiously released in the TME. These molecules play important roles in establishing the strictly tuned relationship between tumor and stromal cells whose balance is critical for tumor development and progression, as will be discussed in the following sections of this review. Moreover, this analysis led us to identifying many Class III OAs that belong to signal transduction pathways reported to be deregulated in breast and other cancers, such as mitogen activated protein kinase (MAPK) [33], Survivin [34], Aurora kinase [35], and* src *pathway molecules [36]. It is worth noting that some of these networks seem to be regulatory keys of therapeutic resistance, such as Survivin [37], Topoisomerase II *α* [38], Desmoglein 2 [39], BCL2-interacting killer [40], and ribonucleotide reductase M2 polypeptide [41]. In addition, several identified proteins have a role in CSC self-renewal, which has been demonstrated in the cases of maternal embryonic leucine zipper kinase [42], transcription factor AP-2 *γ* [43], the microtubule associated TPX2 protein [44], and Aurora kinase A [44]. At present, our efforts are focused on the characterization of some of these targets and our final goal is the setup of new DNA vaccines that will be tested in BALB-neuT mice in association with anti-HER2 vaccination, in order to improve the vaccination's efficacy against advanced tumor and metastases. A more detailed analysis of OAs that are selectively expressed by the various populations that constitute TME may end up providing us with a sort of tumor Rosetta Stone which could help unveil the reciprocal connection between tumor, CSCs, and stroma.

As reported in several clinical studies, the expression of noncoding genes, such as microRNAs (miRNAs), correlates with cancer relapse and metastasis formation [45]. Several miRNAs contribute to tumor progression in virtue of their ability to posttranscriptionally modulate the expression of oncogenes or oncosuppressors. They can act directly on TME, regulating both the survival of more differentiated cancer cells and the maintenance of a CSC phenotype [46] and controlling neoangiogenesis during tumor progression [47]. Results from experimental studies, which have been strengthened by the human cancer miRNA expression profile, have led researchers to the identification of miRNAs as potent regulators of the crosstalk between cancer and stromal cells [48]. Even if miRNAs cannot be considered oncoantigens because of their lack of immunogenicity, the identification of miRNAs, which are differentially expressed in the tumor, can lead to the identification of their target genes as potential oncoantigens or oncosuppressors, nevertheless [19].

Of note among the miRNAs that have recently been identified is the strong upregulation of miR-135b which has been found in invasive mammary BALB-neuT carcinomas; acting on its targets, midline 1 (MID1) and mitochondrial carrier homolog 2 (MTCH2), it regulates CSC stemness* in vitro* and cancer cell metastatization* in vivo *[49]. This newly unveiled role for miR-135b in mammary carcinogenesis, as observed in other tumors such as colon cancer [50], osteosarcoma [51], ependymoma [52], and hepatocellular carcinoma [53], can provide the basis for the exploration of miR-135b, MID1, and MTCH2's potential as new therapeutic targets in mammary carcinogenesis.

## 3. CSCs on Stage

The scientific spotlight has very recently been pointed on CSCs, the subpopulation of cells endowed with self-renewal potential and refractoriness to chemo- and radiotherapy that are capable of sustaining tumor growth and progression by giving rise to the heterogeneous population of tumor cells found within a tumor [54]. Even though the initial idea of CSCs as static entities [55] has been overtaken [56], it is well accepted that they control cancer development and progression in a manner that is guided by environmental factors [57]. CSCs are thought to reside in a highly specialized niche that is made up of stromal, endothelial, and more differentiated tumor cells that stimulate CSC survival and stemness via cell to cell contact, paracrine, and other signals [58]. A central role is played here by interleukin- (IL-) 6, which is produced by CSCs and noncancerous cells, MSCs, and immune cells. IL-6 promotes CSC self-renewal, the recruitment of MSCs and immune cells, and the preservation of an inflammatory state that favors tumor growth. Moreover, IL-6 promotes the conversion of more differentiated tumor cells into CSCs, inducing the epithelial-to-mesenchymal transition (EMT). Recently, it has been shown that HER2 overexpression in breast CSCs increases IL-6 secretion [59] which is involved in Trastuzumab resistance [60].

We have recently demonstrated that an autocrine loop involving toll-like receptor 2/high mobility group box-1/NF*κ*B (TLR2/HMGB1/NF*κ*B) induces the enhanced secretion of vascular endothelial growth factor (VEGF) and IL-6 in Sca1^+^ [61] CSCs, derived from BALB-neuT TUBO cells, that in turn activates the transcription factor signal transducer and activator of transcription 3 (STAT3), thus promoting CSC self-renewal [62]. This pathway also induces the secretion of transforming growth factor- (TGF-)*β*, a cytokine that induces EMT and the secretion of matrix components that favor metastatization [63]. Moreover, TGF-*β* recruits endothelial cells and promotes their proliferation, enhancing angiogenesis [64]. Therefore, HER2 positive CSCs promote their own self-renewal, by upregulating TLR2 and secreting its endogenous ligand HMGB1, and generate a favorable microenvironment for tumor progression. This is a very important observation since HMGB1 is not only secreted by CSCs but also secreted by activated dendritic cells (DCs) [65] and necrotic cells [66] and thus is one of the most important molecules driving tumor escape from cytotoxic treatment.

IL-6 stimulates CSCs, MSCs, and fibroblasts and causes them to secrete IL-8, another key cytokine that promotes CSC self-renewal. It is worth noting that HER2 positive CSCs overexpress IL-8 receptors CXCR1/2 [67], which in turn induce HER2 phosphorylation and the activation of its downstream signaling pathway, generating a positive feedback mechanism that promotes CSC expansion [68]. The inhibition of CXCR1, either by mAbs or specific inhibitors, reduces CSC self-renewal, induces cell apoptosis, and inhibits metastatization in breast cancer, indicating that this receptor may be a promising target for combined anticancer therapies [69]. Similar IL-6-dependent upregulation is observed in the chemokine (C-C motif) ligand (CCL) 2 (also known as monocyte chemotactic protein-1, MCP-1), whose production is induced by IL-6 in both tumor cells and stromal cells and that supports the expansion of the CSC compartment by activating the Notch1 signaling pathway [70]. We demonstrated, by microarray analysis, that CCL2 expression increases in BALB-neuT mice as carcinogenesis progresses [71], and its causal role in cancer development was further supported by the observation that BALB-neuT mice, which were knocked-out (KO) for CCL2, displayed prolonged survival over BALB-neuT mice wild-type (WT) for this chemokine [72].

The characterization of all the cytokine networks that connect CSCs, tumor cells, and stromal cells may pave the way for new therapeutic strategies and provide diagnostic and prognostic markers for patients. In this regard, many clinical studies have shown that high serum levels of IL-8 and IL-6 correlate with poor prognosis in breast cancer patients [73, 74]. Therefore, the design of specific cytokine receptor inhibitors and the assessment of their efficacy in clinical settings may be a source of great potential for future research.

## 4. Fighting against Proangiogenic OAs

Vascular ECs thoroughly govern angiogenesis, a process that supports the growth of many kinds of solid tumors including breast cancer, providing nutrients and oxygen to proliferating cells, thereby allowing cancer cells to invade tissues and develop metastases. Tumor cells have been observed to preferentially align towards and associate with ECs, even prior to the angiogenic switch [75]. Thanks to this strategic tidiness, ECs and tumor cells can bidirectionally communicate through a complex network of both soluble and insoluble signaling molecules that drive cellular differentiation and find ways to foster the tumor. Moreover, ECs are the most important interface between circulating blood cells, tumor cells, and extracellular matrix and play a pivotal role in controlling leukocyte recruitment and tumor cell behavior during angiogenesis.

A great deal of effort has been poured into attempts to block tumor angiogenesis. In this respect, VEGF-A is nowadays the most renowned therapeutic target. The interaction between VEGF ligands and their EC expressed receptors stimulates angiogenesis and promotes EC permeability, survival, migration, and the invasive potential of cancer cells [76]. Bevacizumab is a recombinant humanized mAb developed against VEGF-A [77], which has been broadly studied in phase III clinical trials and is now FDA-approved for the treatment of metastatic colorectal cancer, nonsmall cell lung cancer, and breast cancer [78]. Other drugs that inhibit the tyrosine kinase activity of VEGFRs, like sunitinib [79], sorafenib [80], axitinib [81], pazopanib [82], vandetanib [83], cabozantinib [84], tivozatinib [85], and linifanib [86], have been developed. Sorafenib has been approved for the treatment of unresectable hepatocellular carcinoma and advanced renal cell carcinoma (RCC), whereas sunitinib has been approved for the treatment of gastrointestinal stromal tumors and metastatic RCC, but only modest benefit has been observed in other types of cancer [87].

Despite many steps forward in the setup of antiangiogenic protocols being made, the development of tumor resistance and the occurrence of relapse in a high percentage of patients have prompted clinicians and researchers to join forces and find new targets for the development of more efficacious therapies. For these reasons, the immune-targeting of OAs expressed on ECs seems to be a successful direction to move towards. As described below, we have tested various DNA vaccination strategies that target tumor angiogenesis; all these vaccines have demonstrated high efficacy without any toxic effect, further stressing the therapeutic potential of targeting tumor ECs in HER2 positive tumors.

Of the class II OAs found to be overexpressed in tumor ECs during BALB-neuT cancer progression [88], the most promising is angiomotin (Amot), a member of the Motin protein family. Using a construct that encodes the kringle domains 1–4 of angiostatin to screen a yeast two-hybrid placenta cDNA library for angiostatin-binding peptides [89], Amot was originally identified as one of the angiostatin receptors. Amot is normally expressed on ECs, where it exerts its proangiogenic activity and stimulates EC migration during angiogenesis [90]. Amot is overexpressed compared to normal tissues in human breast tumors and its presence correlates with poor prognosis and metastatic disease [90]. These findings suggest that Amot has an important role to play during breast tumor progression and may be an optimal target for anticancer therapy [91]. In virtue of these features, we decided to elicit an immunological response against Amot, by means of DNA vaccination, in mice that bear microscopic invasive mammary cancers. This strategy was successfully applied in BALB-neuT mice as well as in the PyMT mouse model of breast cancer, in which carcinogenesis is driven by the polyoma middle T oncoantigen [92]. The therapeutic effect of anti-Amot vaccination was mediated by the induction of specific antibodies that induced increased tumor vessel permeability, which, in turn, resulted in both an increase in chemotherapy efficacy and major epitope spreading, which was accompanied by the induction of a specific anti-HER2 antibody response that further contrasted tumor growth [93].

Another member of the Motin family, angiomotin-like 1 (AmotL1), is an attractive target for antitumor interventions. AmotL1 is endowed with proangiogenic properties that affect EC polarization, directional migration, and the stability of tight junctions during angiogenic sprouting; it may compensate for the absence of Amot and vice versa [94]. Even though our preliminary data indicate that DNA vaccination against AmotL1 is not effective in the prevention of mammary tumor appearance in BALB-neuT mice, encouraging data have come from a combined DNA vaccine against HER2 and AmotL1. Even more promising results have been obtained using a combined DNA vaccine against HER2, Amot, and AmotL1 (Barutello G et al., unpublished data). This kind of vaccination exploits the synergistic effect which stems from the combined action of antibodies which target both the ECs of neoformed tumor vessels and the tumor cells themselves.

Membrane-bound KitL (mbKitL), which is involved in the c-Kit/KitL system required for tumor angiogenesis [95], is an additional promising target for antiangiogenic cancer immunotherapy. mbKitL is expressed on tumor ECs and is essential for providing them with survival signals, as is clearly exploited in the role that c-Kit signaling network plays in maintaining breast cancer cells [96]. A DNA vaccine that targets mbKitL is able to inhibit the growth of a mouse HER2 positive transplantable tumor; vaccination impairs tumor vessel formation and stabilization and thus interferes with tumor cell-derived VEGF bioavailability [97].

Besides representing good targets for anticancer therapies, antigens expressed on tumor ECs may also be exploited for tumor diagnosis. In this context, we have recently demonstrated that both ECs and cancer cells in mammary tumors arising in BALB-neuT mice express *α*_*v*_ *β*_3_ integrin, a receptor for several extracellular matrix proteins which harbor an arginine-glycine-aspartic acid (RGD) sequence [98]. *α*_*v*_ *β*_3_ integrin is widely considered to be a marker of the angiogenesis, tumor progression, and invasion of different types of cancer. Since its level of expression correlates with cancer progression [99], we have developed a probe for the optical imaging detection of *α*_*v*_ *β*_3_ integrin and have shown that it can successfully detect microscopic* in situ* carcinomas in BALB-neuT mice, therefore proving itself to be a promising tool for the early diagnosis of breast cancer [98].

## 5. The Controversial Role of Inflammation and Immune Cells in the TME

Despite the fact that natural immune surveillance mechanisms are activated during the early stages of BALB-neuT carcinogenesis [100–103], tumors finally acquire the three immune hallmarks required to progress: the ability to thrive in a chronically inflamed TME, to suppress immune reactivity, and to evade immune recognition [104]. The fight between natural immune surveillance mechanisms and these acquired capabilities is mirrored by the important, yet controversial, role that immune cell infiltrates play in the TME. The tumor stroma of BALB-neuT mice is infiltrated by CD4 and CD8 T lymphocytes and a few B, natural killer (NK), and *γδ* T lymphocytes, but mostly by regulatory T (Tregs) cells, myeloid derived suppressor cells (MDSCs), and tumor associated macrophages (TAMs) that are recruited into TME in response to inflammatory molecules and cytokines being released in the tumor milieu [105, 106].

The acquired ability of BALB-neuT tumors to thrive in a chronically inflamed microenvironment has been highlighted by microarray analyses that have shown the occurrence of an upregulation in four transcriptional networks, in advanced as compared to preneoplastic lesions, whose hub genes code for proinflammatory cytokines IL-1*β*, tumor necrosis factor- (TNF-)*α*, interferon- (IFN-)*γ*, and CCL2 [71]. The final outcome of the activation of these four networks is tumor promotion; however, how each individual network influences tumor progression is neither simple nor unequivocal. For instance, increased IFN-*γ* release in TME during tumor progression appears to play a major tumor inhibitory role and is a marker of the M1 TAMs that express immunostimulatory, antiangiogenic, and tumoricidal functions [107]. Accordingly, IFN-*γ* KO BALB-neuT mice display faster tumor progression, associated with a more intense tumor angiogenesis [71, 108, 109]. Moreover, chronic systemic administration of recombinant IL-12 in BALB-neuT mice induced high and sustained IFN-*γ* production, as detected in the sera of treated mice that in turn caused a delay in tumor onset and a reduction in the number of mammary glands affected by the tumor [109, 110]. The role that the other three networks play in tumor progression is the opposite. They can initially show antitumor activity, but the incipient tumor soon uses them to provide itself with a shortcut for progression. In reality, the activation of CCL2 is directly associated with enhanced progression [72], as discussed above. Similarly, increases in IL-1*β* and TNF-*α* in TME may favor cancer progression either directly [71, 111] or by recruiting suppressor cells [112, 113].

A tumor's ability to exploit inflammation to its own benefit is strictly related to the second immune hallmark of cancer, the capability to suppress the immune response directly or via the recruitment of suppressor cells [104]. IL-1*β* released by stromal cells together with other tumor-derived factors, including granulocyte macrophage colony-stimulating factor (GM-CSF), cyclooxygenase 2 (COX-2), IL-6, and VEGF, induce the accumulation and expansion of MDSCs [112, 113] by triggering Janus kinase (JAK)/STAT3 pathways [114]. MDSCs are a phenotypically heterogeneous population with an immunosuppressive capacity that are, in normal conditions, generated from the bone marrow and rapidly differentiates into mature DCs, macrophages, or granulocytes, while, in cancer bearing patients, present a partial block of maturation [115]. In BALB-neuT tumors, VEGF was detected in the supernatant from primary tumor cultures and from tumor cell lines as well as in the sera of BALB-neuT tumor-bearing mice. A possible explanation may lie in the increase of matrix metalloproteinase- (MMP-) 9 within the tumor mass, as previously shown [116], that mediates the release of growth factors, such as VEGF, stromal cell-derived factor- (SDF-) 1, and mbKitL [117]. Accordingly, any interference with VEGF or mbKitL activity, besides hampering the angiogenic process [97, 118], has been reported to induce MDSC shrinkage [97, 119].

MDSCs exhibit immunosuppressive functions that occur via multiple mechanisms, such as inducible nitric oxide synthase (iNOS) and arginase-1 (Arg-1) production, which suppresses the T-cell immune response in TME via the release of nitric oxide and reactive oxygen species that cause T cell receptor (TCR) nitration and T cell apoptosis and the depletion of L-arginine required for T cell functions [120]. As indoleamine-2, 3-dioxygenase (IDO) appears to be involved in MDSC-mediated T cell inhibition [121] and cyclooxygenase- (COX-) 2 is required to induce, via prostaglandin E- (PGE-) 2, Arg-1 expression by MDSCs [122, 123], a considerable amount of effort is going into inhibiting these molecules [124]. In this respect, we are testing a therapeutic protocol that consists of the concomitant administration of anti-HER2 DNA vaccines and plasmids that code for IDO [125] or COX-2 or short hairpin (sh)RNAs in BALB-neuT mice [24].

In order to curb the significant MDSC contribution to suppressing the immune system, we have looked for additional targets that these cells express both in tumor bearing mice and in cancer patients. As discussed above, B7-H4, a member of the B7 family, has been identified as being overexpressed in BALB-neuT mouse invasive lesions and appears to be an excellent target candidate, thanks to its critical role in the regulation of antigen specific immune responses [3]. Indeed, within TME, the expression of B7-H4 by tumor cells and MDSCs seems to be involved in the inhibition of the T cell response to tumor associated antigens [126]. In the light of these considerations, we are developing DNA plasmids that code for both HER2 and B7-H4 shRNAs, and we propose an evaluation of their efficacy in the inhibition of mammary carcinogenesis (Macagno M, unpublished data). Another important pathway that contributes to tumor mediated immune suppression is found in the CD28 family member, programmed death 1 (PD-1) and its ligand PD-L1 [127]. PD-L1 is expressed by both MDSCs and tumor cells [128] and its interaction with activated T cell expressed PD-1 promotes T cell tolerance by suppressing their cytotoxic capacity and cytokine secretion [127]. We were among the first to show that the PD-1 blockade results in an increased response to antitumor vaccination. In these experiments BALB-neuT mice were vaccinated against HER2 and concomitantly treated with the administration of anti-PD-1 mAb BAT [129].

In response to IL-1*β* stimulation, MDSCs also produce the suppressive cytokine IL-10 [130] which acts on TAMs inducing their reprogramming and polarization towards an M2 phenotype. M2 TAMs support tumor progression through the release of immunosuppressive (i.e., CCL2 and IL-10), proangiogenic (i.e., IL-8 and VEGF), and tissue remodeling (i.e., MMP-2 and MMP-9) factors. Their expansion in breast cancer tissues has been correlated with poor prognosis [131]. In BALB-neuT mice M2 TAMs are the main tumor infiltrating population [105]. The administration of zoledronic acid to BALB-neuT mice can revert M2 polarization by interfering with the mevalonate pathway and thus hamper IL-10 and VEGF production, recovering the release of IFN-*γ* in the mammary glands of treated mice [105].

HER2 and the other OAs expressed by mammary tumors in BALB-neuT mice are self-molecules toward which the immune system is tolerant [132]. As a consequence, the predominant effector T-cells in the TME are presumably constituted of low avidity OA-specific T cells whose activity is inhibited by Tregs that first expand in the spleen and tumor draining lymph nodes during cancer progression and in TME in later phases [100, 132, 133]. This situation reproduces what normally happens in tumor bearing patients [134] and is part of the ability to suppress immune reactivity that the tumor acquires during progression [104]. Indeed, natural immune surveillance somehow counteracts Treg expansion in the early phases of carcinogenesis in BALB-neuT mice. In complement C3 KO BALB-neuT mice, tumor progression occurs earlier and this is associated with the increased expansion of Treg cells over complement competent BALB-neuT mice [102]. This increased Treg expansion is prompted by a lack of C3a and C5a, whose receptor signaling is required during the early events of effector T cell activation [135] and negatively modulates Treg function by inducing FoxP3 downregulation [136]. Its absence in BALB-neuT C3 KO mice deflects naïve T cells into Treg [137] and potentiates their function [136].

The down modulation of MHC class I (MHC I) [138] is the mechanism most frequently exploited by tumor cells to escape from immune recognition [139]. It is intriguing that an inverse correlation exists between HER2 overexpression and the expression of MHC I and of the components of the antigen-processing machinery [140]. MHC I down modulation, albeit incomplete, means that cancer cells are more susceptible to NK cell-mediated lysis, if NK receptor activating ligands are present. This may have an impact on cancer progression at least in the initial stages of carcinogenesis. The fundamental role that NK cells play in hampering the expansion of incipient BALB-neuT tumors has been investigated in perforin (PFP) KO BALB-neuT mice, as the majority of NK mediated protection relies on the release of PFP on target cells. In fact, both female [103] and male [141] BALB-neuT PFP KO mice show fourfold increases in mammary carcinoma incidence. Nevertheless, preliminary results also indicate that advanced BALB-neuT tumors downregulate the expression of ligands that activate NK receptors (Lanzardo S, unpublished data), suggesting that advanced tumors reach a balance between a loss of sensitivity to CD8^+^ T cell killing and the maintenance of NK-cell-inhibitory specificities. We are now evaluating the expression of MHC I and of some NK ligands in TUBO-derived CSCs to assess whether NK cells recognize and more efficiently kill CSCs than their differentiated counterparts, as has already been shown for colon cancer-derived CSCs [142].

## 6. Role of Adipocytes and Fibroblasts in Breast Cancer Progression

While immune cells are well recognized as major players in the orchestration of a permissive TME, other cell populations have only recently been recognized as active parts of the tumor promoting ability of TME. These include CAAs and FACs.

Besides its classical definition as a fat reservoir, adipose tissue is now considered to be a fully functioning endocrine organ [143] that secretes growth factors and cytokines, known as adipokines, which are involved in angiogenesis, immunity, and endocrine signaling [144]. Adipocytes enshroud the mammary gland, regulating epithelial cell growth during the hormonally controlled courses of mammary gland development, from pubertal maturation to involution after lactation [145].

The understanding of the important, but still underestimated, role of adipocytes in cancer stems from several studies which highlight the anatomical proximity of many tumors to adipose tissues and point to the positive correlation between obesity and higher cancer risk [146–149]. Adipocytes can, under the pressure of cancer cell stimuli, abdicate their physiological role in favor of tumor promoting activities in breast cancers that grow in an adipose tissue dominated context. In this way they become CAAs that exhibit decreased lipid content, reduced adipocytes marker expression, and an overexpression of proinflammatory cytokines and MMPs, such as MMP-11 and MMP-9 [150, 151]. It is worth noting that MMP-9 has been identified as being overexpressed in BALB-neuT mammary cancer which would seem to point to its important role during tumor progression.

A number of studies have shown that CAAs support and expedite breast cancer progression [152–154] by providing proinflammatory cytokines, such as IL-6, TNF-*α*, and reactive oxygen species [155]. On the other hand, IL-6 in breast TME seems to stimulate the proinvasive effects of CAAs, besides promoting CSC self-renewal as discussed above [150]. Moreover, CAAs in TME can differentiate in fibroblast-like cells that, together with other stromal cell populations, participate in the generation of dense collagenous stroma, the so called desmoplastic response, typically observed in breast cancer [156].

CAAs functions are mainly mediated by leptin and adiponectin, two functionally opposite members of the adipokine family, that seem to play a pivotal role in cancer progression [157]. Leptin promotes tumor growth, eliciting the activity of several signaling pathways such as insulin-like growth factor-1 (IGF-1) and HER2 and inducing the expression of MMP-2, MMP-9, and VEGF, which finally promote cell migration and metastatic spreading [158, 159]. Furthermore, leptin exerts a chemoattractant effect on macrophages and monocytes [160] and stimulates them to produce the inflammatory cytokine TNF-*α* that in turn manifests proangiogenic activity [161]. On the other hand, adiponectin acts as an antiangiogenic and anti-inflammatory factor that is able to repress proliferation and induce apoptosis in breast cancer cells [147, 162]. Interestingly, some studies have found that caloric restriction can exert an anticancer effect via alterations in systemic IGF-1 and NF-*κ*B levels [163].

Altogether these data suggest that the recently discovered therapeutic potential of adipocytes could open new and promising perspectives in breast cancer treatment. One example of this comes from the preclinical experience gained with adipokine osteopontin (OPN), also called “early T cell-activation gene 1,” a multifunctional component of the extracellular matrix that has been linked to a plethora of autoimmune diseases [164]. OPN has very recently been rediscovered as a diagnostic and prognostic marker in HER2 positive breast cancer [165] and one whose abnormal expression in patients is linked to poor prognosis [166]. It has also been proposed that the autocrine production of OPN by tumor cells may be an important factor that allows invasion and survival to occur [167]. In fact, the interaction between extracellular matrix deposited OPN and cell adhesion molecules, such as *α*_*v*_ *β*_3_ integrins which are overexpressed in BALB-neuT tumors [98], increases both the expression of VEGF in ECs, allowing neovascularization, and the activation of connective tissue growth factor and cysteine-rich angiogenic inducer 61(CYR61), which enhances neovascularization and mammary tumor growth* in vivo *[168].

As previously mentioned, CAAs can differentiate into fibroblast-like cells that share many properties with CAFs [169]. CAFs promote tumor growth and invasion secreting proangiogenic factors (i.e., VEGF-A and MMP-9) [170], proinflammatory molecules (i.e., SDF-1, IL-6, and IL-1*β*) [171], and several growth factors (i.e., TGF-*β*, platelet-derived growth factor, PDGF, and basic fibroblast growth factor, bFGF) [172, 173]. In particular, the aberrant production of IL-6 and CCL2 in mammary cancer activates STAT3 in CAFs, which finally sustains tumor-associated inflammation and is required for breast cancer cell migration [174]. Certainly, in BALB-neuT mice this network seems to be particularly interesting, as in a BALB-neuT mice knock-in for a constitutively active Stat3* allele*, we observed an earlier and more invasive onset of mammary tumors [175].

## 7. MSCs Are Key Players in the TME Orchestra

Adult multipotent MSCs make for a fascinating TME population which is able to control the interplay between cancer cells and tumor stroma. Physiologically, MSCs are located predominantly in the bone marrow and contribute to the maintenance and regeneration of a variety of connective tissues [176]. During injury and inflammation, they are recruited to damaged sites via the release of soluble molecules and operate in tissue remodeling [177].

MSCs also localize into different types of solid tumors which they first migrate towards then integrate into the tumor-associated stroma [178]. Recent studies have provided direct evidence that MSCs are recruited in TME by a broad range of soluble factors which are secreted by cancer cells and CSCs, including IL-6 [179], VEGF and bFGF [180], CCL2 [181], SDF-1*α* [182], and HMGB1 [183]. Moreover, stressful conditions, such as irradiation [184], hypoxia [185] and, cellular damage [183], can enhance the recruitment of MSCs to the site of growing tumors. Once there, MSCs contribute to the development of an active TME, in which bone marrow-derived MSCs generate CAFs, while local adipose tissue-derived MSCs contribute mainly to the vascular and fibrovascular stroma (pericytes, myofibroblasts, and ECs) [186]. In addition, MSCs interact with tumor cells and with all other stromal cells through a broad range of signaling molecules, generating complex crosstalk whose net effect is to stimulate tumor progression. For example, MSCs can promote breast cancer neoangiogenesis, possibly thorough the secretion of macrophage inflammatory protein 2 (MIP-2), VEGF, TGF-*β*, and IL-6 [187] and display potent immunomodulatory properties [188] that enable them to inhibit CTLs and NK cells by stimulating Tregs through the release of TGF-*β*1 [189].

Conflicting data have led to the hypothesis that two opposing immunological MSC phenotypes exist, one proinflammatory and one immunosuppressive, which are dependent on the engagement of specific TLRs [190]. The role of TLR2 is still debated, with some studies claiming that TLR2 activation on MSCs inhibits their immunosuppressive properties [191], while others argue that TLR2 stimulation does not affect this capability [192]. Notably, these considerations are mostly based on* in vitro* experiments. Therefore, BALB-neuT mice may well be a suitable tool for the difficult task of definitely clarifying the role of TLR2 in MSCs. Starting from our observation that TLR2 drives mammary CSC self-renewal [62], we are developing BALB-neuT mice that are KO for TLR2, in which we would like to characterize the role of TLR2 not only in CSCs but also in MSCs and other stromal populations.

MSCs are thought to contribute to CSC niche generation, thus regulating cancer cell stemness through multiple pathways and secreted factors (i.e., IL-6 and CXCL7 [193], PGE-2 [194], EGF, bFGF, bone morphogenic protein (BMP) 4, TGF-*β*1, SDF-1*α*, and CCL5 [195], among others) that increase CSC self-renewal and expand the CSC population.

Furthermore, MSCs promote various malignant features; they control the metastatic ability of breast cancer cells by inducing EMT through the secretion of PDGF-D [196], TGF-*β*1 [197], IL-6, and VEGF [198] and promote cancer cell migration through the release of a plethora of chemokines such as CCL5 [199], CXCL1 and CXCL5 [200], CXCL9, CXCL10, and CXCL11 [201] or SDF-1 [202]. For all these reasons, MSCs represent an attractive target when considering the design of new and promising anticancer treatments. However, the lack of specific markers that discriminate MSCs from other cell types makes the direct targeting of the MSC population an unrealistic approach. An attempt to disrupt signaling pathways between MSCs and CSCs is more feasible. In fact, the experience we have gained with the BALB-neuT model suggests that some of the molecules released by MSCs, such as IL-6, TGF-*β*, and HMGB1, are key molecules in CSC self-renewal and cancer progression [62]. The targeting of these molecules or their receptors, which are somehow redundant in different malignant processes, may be a means by which to interfere with tumor pathogenesis on multiple levels.

In recent years, there has been growing interest in the use of MSCs as a tool for the target-specific delivery of therapeutic agents, because their avid tumor tropism means that they can act as a sort of Trojan horse. MSCs can be genetically engineered to express antitumor cytokines, such as IFN-*β* [203], IL-12 [204], and TRAIL [205], or prodrugs such as cytosine deaminase [206], which are then released directly into the tumor milieu, thus greatly reducing their systemic toxicity. These approaches have been shown to be effective in the management of various preclinical tumor models. However, these killer MSCs may still maintain all the protumoral features here described and some concerns still exist about the potential conversion of MSCs into cancer cells themselves [207]. Therefore, the actual exploitation of MSCs as a tool for anticancer therapy still needs more study, and BALB-neuT mice represent a good model through which to evaluate the feasibility of this approach, in the context of HER2 positive breast cancers.

## 8. Conclusions

The growth and progression of breast cancer cells depend not only on their intrinsic malignant potential but also on a mutual and continuous dialogue between cancer cells and stromal, immune, and endothelial cells within TME. Multidirectional interactions between several substances, such as cytokines, MMPs, and growth factors, secreted by all these populations closely cooperate for the generation of a permissive TME that is crucial for successful cancer progression. This complex and finely tuned interplay between cancer and stromal cells during breast cancer development is summarized in Figure 1.

Experimental studies, conducted on preclinical models, have provided significant hints as to how TME affects tumor progression and response to therapy. BALB-neuT mice are an emblematic example in this regard. Over the years, the exploitation of this model has allowed the identification of novel molecular targets to be carried out and has prompted us to develop new, promising therapeutic approaches. On the other hand, it has provided evidence that the direct targeting of cancer cells is not enough to obtain complete disease remission. This highlights the need to extend antitumor intervention beyond the tumor bulk, as targeting both cancer cells and other TME cell populations may be a more complete and effective strategy.

Given the significant role that CSCs play in the various steps of tumor development and TME modulation, we have recently focused on the identification of pathways that regulate CSC self-renewal and influence, on TME as well as on the investigation of CSC-specific antigens. Another promising field of study can be found in action on tumor angiogenesis; in particular, strategies that modulate vessel permeability may also stabilize tumor vessels and favor both the distribution of traditional drugs into the tumor milieu and immune cell accessibility. As in the BALB-neuT model, the tumor infiltrate is mainly composed of immunosuppressive cells. The addition of immunomodulatory strategies to standard anticancer approaches could be essential for a therapeutic success.

Other TME cell populations, which are still almost unexplored in this model and whose involvement in tumor pathogenesis is still in its infancy, are found in CAAs and CAFs. Given the tissue organization of mammary glands and of the tumor within, which is rich in adipose cells and fibrous tissue, the identification of markers that are overexpressed by CAFs and CAAs may lead to the eradication of these cells which favor cancer progression through the production of various cytokines and extracellular matrix proteins. The blockade of these soluble molecules or their receptors may be an interesting option, as the disruption of the TME signaling network may make cancer cells themselves more amenable to traditional approaches. The drugs used in these combined treatments may be successfully delivered to TME by exploiting the avid tropism of MSCs, which may be engineered in order to produce molecules that inhibit the different populations present into the TME.

In conclusion, the targeting of multiple TME populations may represent the best strategy for setting up innovative anticancer treatments that significantly improve patient survival and shrink the development of drug resistance; in this regard, BALB-neuT mice provide a suitable experimental setting, thanks to the high translational value of this model.
